# Supplementary material for: Osteocyte CIITA aggravates osteolytic bone lesions in myeloma
Source: Nat Commun. 2022 Jun 27;13:3684. doi: 10.1038/s41467-022-31356-7 (PMC9237076; doi:10.1038/s41467-022-31356-7)
Supplement: Supplementary file 1 — Supplementary Information [file 41467_2022_31356_MOESM1_ESM.pdf]

SUPPLEMENTAL INFORMATION

Osteocyte CIITA Aggravates Osteolytic Bone Lesions in Myeloma

Huan Liu, Jin He, Rozita Bagheri-Yarmand, Zongwei Li, Rui Liu, Zhiming Wang, Duc-hiep Bach, Yung-hsing Huang, Pei Lin, Theresa A. Guise, Robert F. Gagel, and Jing Yang

Supplementary Figures

Supplementary Tables

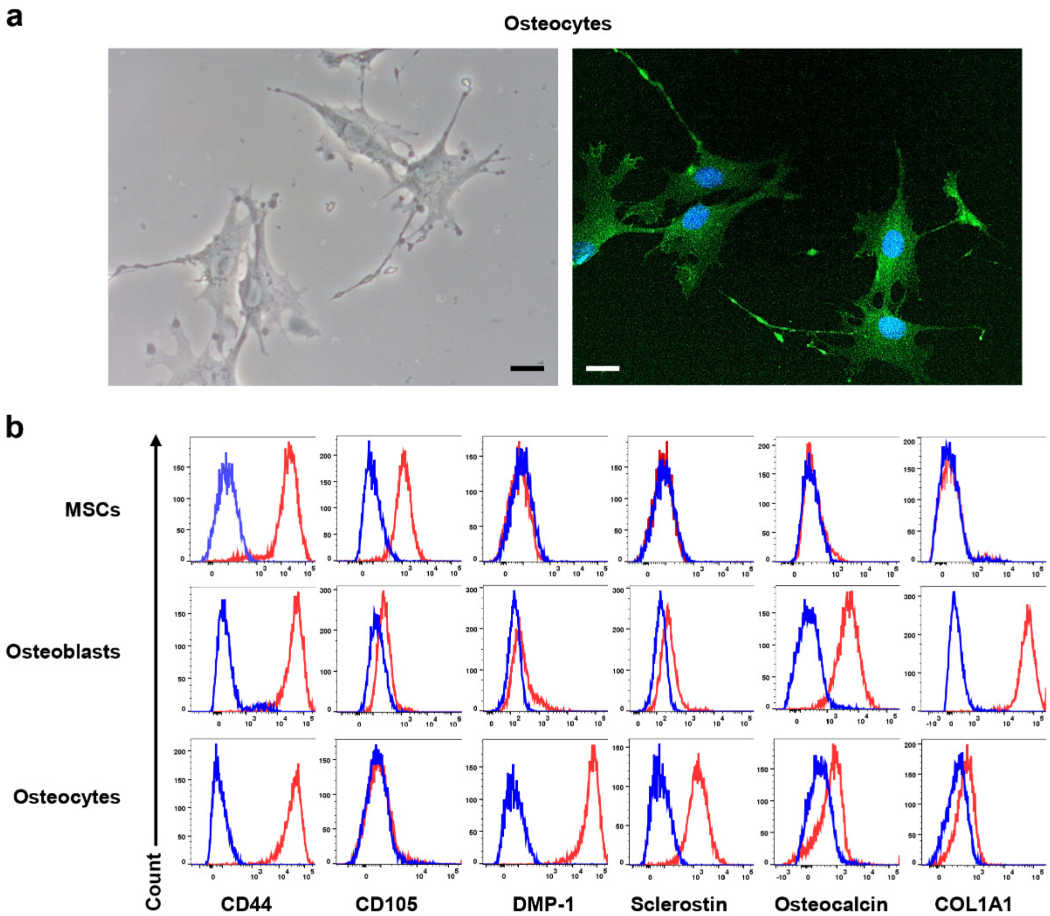

Supplementary Fig. 1. Characterization of osteocytes. Cells were isolated from the femurs of C57BL/6 mice**. a** Representative images show the morphology of primary osteocytes after 7 days of culturing under light microscopy (left) or staining for E11/GP38 under fluorescent microscope (right). Scale bar: 20 µm. Images shown are representative of three independent experiments. **b** Identification of MSCs, osteoblasts, and osteocytes using flow cytometry. Shown are representative histograms of the surface markers for MSCs (CD44 and CD105), osteocytes (DMP-1 and sclerostin), and osteoblasts (osteocalcin and COL1A1). Blue lines indicate isotype controls. Data shown are representative of two independent experiments.

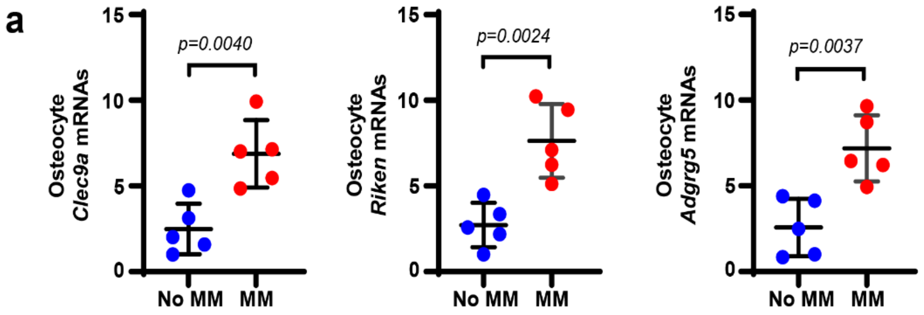

b

	BV/TV (%)	OS/BS (%)	Ob.S/BS (%)	ES/BS (%)	Oc.S/BS (%)
Clec9a **mRNA**	r=0.0526	r=0.0611	r=0.1355	r=-0.3012	r=0.2569
Riken **mRNA**	r=0.1713	r=-0.2757	r=-0.1005	r=0.1429	r=0.3685
Adgrg5 **mRNA**	r=0.0646	r=-0.2359	r=-0.1026	r=-0.0217	r=-0.0729

Supplementary Fig. 2. The correlation between the expression of candidate genes expressed in osteocytes and the status of bone lesions in myeloma-bearing mice. a qPCR analysis shows the levels of *Clec9a*, *Riken,* and *Adgrg5* mRNAs in the osteocytes isolated from myeloma-bearing or control mice. The data are mean ± SD, n=5 mice/group. *P* value was determined using unpaired two-tailed *t*-test. **b** Correlation coefficients between the levels of *Clec9a*, *Riken,* or *Adgrg5* mRNAs in osteocytes and the percentages of BV/TV, OS/BS, Ob.S/BS, ES/BS, and Oc.S/BS in myeloma-bearing mice (n = 10) and control mice (n = 10). The correlations were evaluated using the Pearson coefficient with two-tailed P value. r, correlation coefficient.

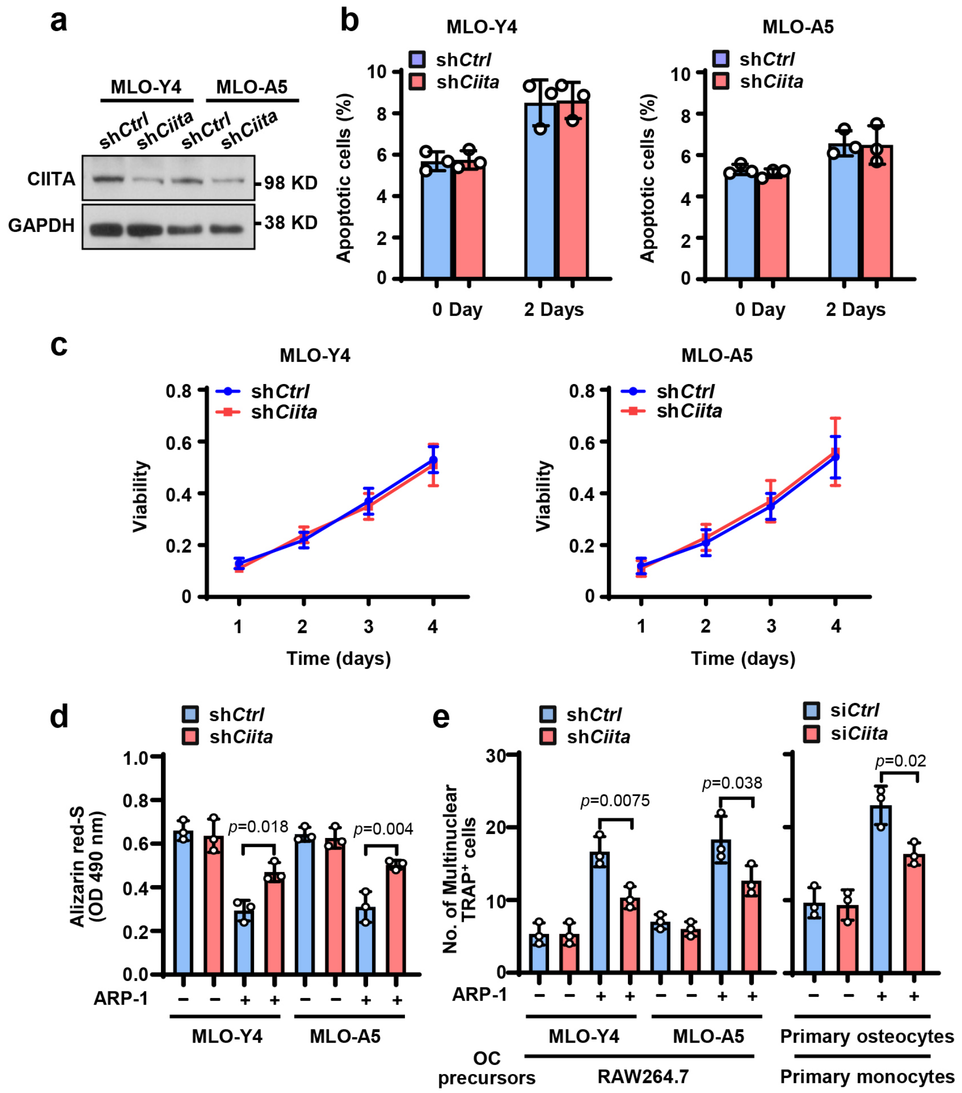

Supplementary Fig. 3. Knockdown of CIITA in osteocytes reduces myeloma-induced enhancement of osteoclastogenesis and inhibition of osteoblastogenesis. a Western blots show CIITA expression in MLO-Y4 or MLO-A5 osteocytes transfected with non-targeted shRNA (sh*Ctrl*) or *Ciita* shRNA (sh*Ciita*). GAPDH levels served as protein loading controls. Data shown are representative of two independent experiments. **b-c** Percentages of apoptosis in 0- or 48-h cultures (**b**) and the viability of sh*Ctrl* or sh*Ciita* MLO-Y4 or MLO-A5 cells in 4-day cultures (**c**). Data shown are mean ± SD, n=3 biological replicates. **d-e** The sh*Ctrl* or sh*Ciita* MLO-Y4 or MLO-A5 cells or si*Ctrl* or si*Ciita* primary osteocytes were co-cultured with or

without myeloma ARP-1 cells for 2 days, and then provided with fresh medium for another 2 days for collecting osteocyte CM. The osteoblast precursor MC3T3-E1 and the osteoclast precursor Raw264.7 or primary monocytes were cultured with the osteocyte CM. After culturing, the cells were subjected to Alizarin red-S staining for osteoblast differentiation or to TRAP staining for osteoclast differentiation. Shown are summarized data from Alizarin red-S staining (**d**) and numbers of multinuclear (≥3) $TRAP^+$ cells (**e**). Data are mean ± SD, n = 3 biological replicates. *P* values were determined using one-way ANOVA with Tukey's multiple comparisons test.

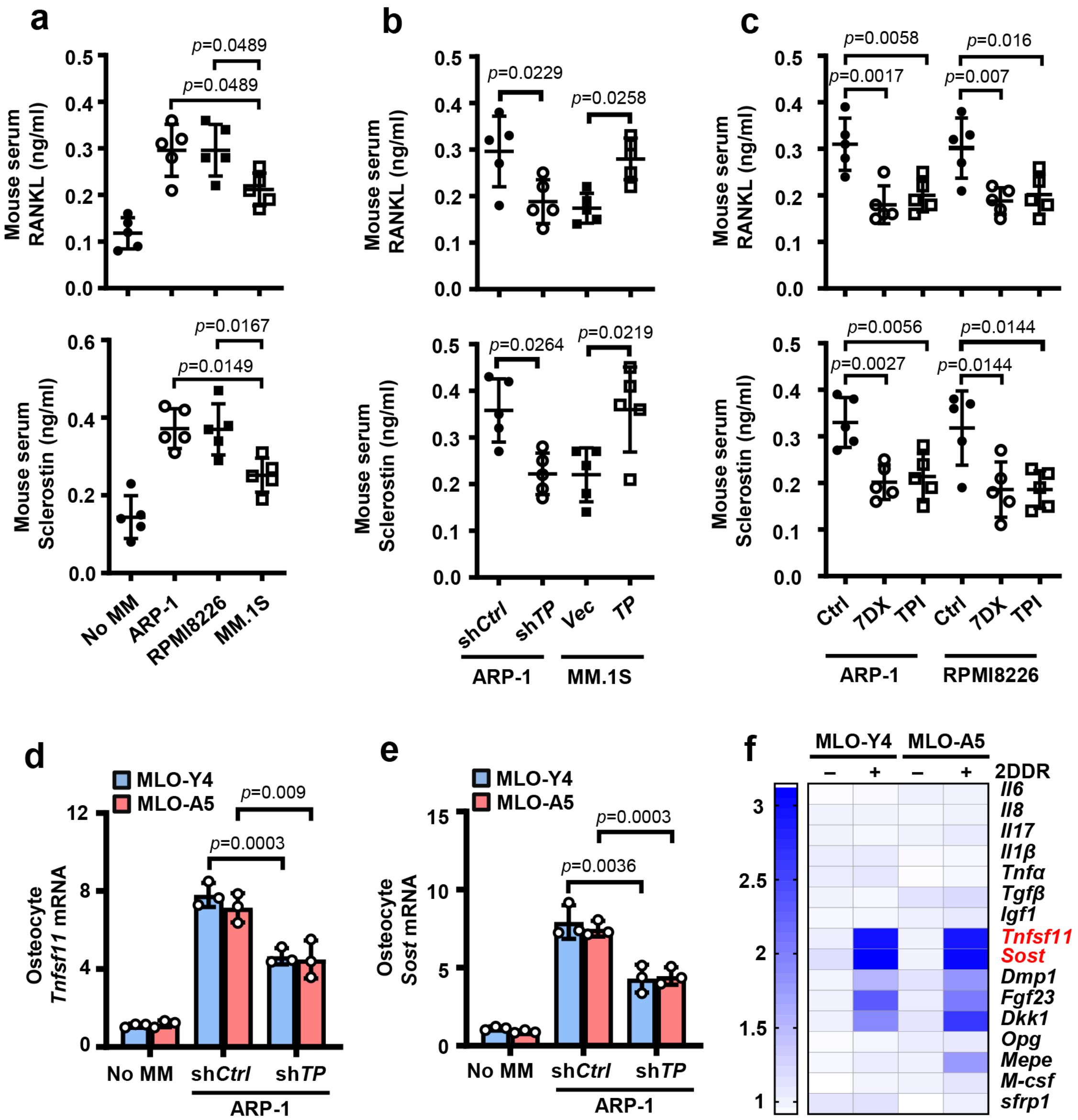

Supplementary Fig. 4. Myeloma TP/2DDR increases RANKL and SOST expression in osteocytes. a The high TP–expressing human myeloma cell lines ARP-1 and RPMI8226 and the low TP–expressing human myeloma cell line MM.1S (5×10^5 cell/mouse) were injected into the femurs of SCID mice. **b** Shown are the levels of RANKL (top) and sclerostin (bottom) in the serum of SCID mice injected with ARP-1 cells expressing non-targeted control shRNAs (sh*Ctrl*) or TP shRNAs (sh*TP*) or with MM.1S cells expressing empty control vector (*Vec*) or TP cDNAs

(*TP*). **c** ARP-1 or RPMI8226 cells were injected into the femurs of SCID mice. After 3 weeks, mice were treated with PBS as vehicle controls (Ctrl) or the TP inhibitor 7DX (200 µg/kg) or TPI (300 µg/kg). After treatment, mouse sera were collected for ELISA analysis. Shown are the levels of RANKL (top) or sclerostin (bottom) in mouse serum. Data are mean ± SD, n = 5 mice/group. **d-e** Osteocyte cell lines MLO-Y4 and MLO-A5 were co-cultured with sh*Ctrl* or sh*TP* ARP-1 cells for 24 h. qPCR analysis showed the relative expression of *Tnfsf11* (**d**) or *Sost* (**e**) in osteocytes. Data are mean ± SD, n = 3 biological replicates. **f** MLO-Y4 or MLO-A5 osteocytes were cultured with 1 mM 2-deoxy-D-ribose (2DDR) for 24 h. Quantitative PCR analysis showed the relative expression of cytokine genes in osteocytes. *P* values were determined using one-way ANOVA with Tukey's multiple comparisons test.

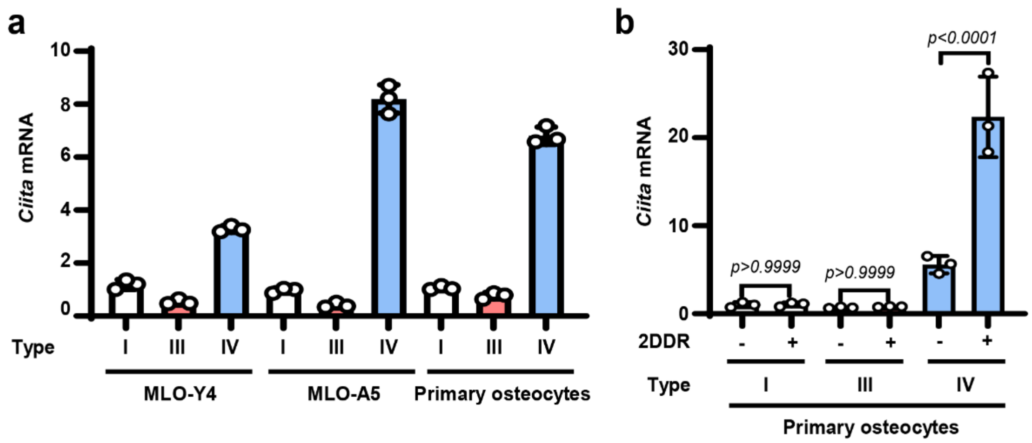

Supplementary Fig. 5. 2DDR enhances the expression of *Ciita* type IV in osteocytes. a Quantitative PCR shows the relative expression of various isoforms of *Ciita* in MLO-Y4, MLO-A5, and primary murine osteocytes. Data are mean ± SD, n = 3 biological replicates. **b** Quantitative PCR shows the relative expression of *Ciita* type IV in primary murine osteocytes that were cultured with or without 1 mM 2DDR for 2 days. Data are mean ± SD, n = 3 biological replicates. *P* values were determined using unpaired two-tailed *t*-test.

Uncropped Figures

Supplementary Fig. 3a

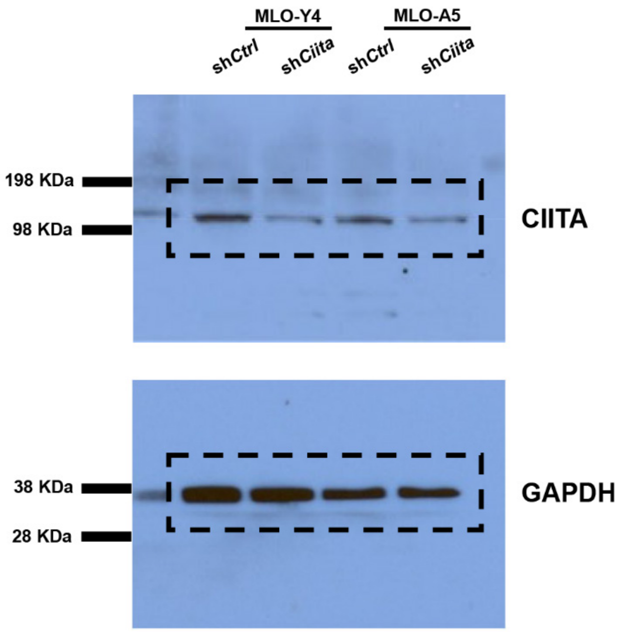

Supplementary Tables

table S1. Identification of AP2α that binds to the CIITA in osteocytes using mass spectrometry.

Gene name	Peptide
CIITA	K.LAWELGR.R
	K.FTIEPFK.A
	K.LLQAAEEK.F
	K.GLVQHPPR.A
	R.GLLAGLFQK.K
	K.SYWAGAVSR.A
	K.SLKDVEDLGK.L
	R.ELATPDWAER.Q
	R.FLAGLIFQPPAR.C
	K.WPEPVEQFYR.S
	K.FTAAGAQQLAASLR.R
	K.RPFPEELPADLK.H
	R.SSSEDTAGELPAVR.D

	R.AALDSPPGALAELAK.L
AP2α	K.VTVAEVQR.R
	K.IGLNLPAGR.R
	K.AVAEFLNR.Q
	R.LSLLSSTSK.Y
	K.EFTDLLAQDR.S
	R.QHSDPNEQVTR.K

Mass spectrum analysis of the upper band.

Protein	Unique peptide	Total peptides	% peptide ratio
**CIITA**	**31**	**55**	**56.36**
KRT1	16	21	76.19
KRT2	15	18	83.33
LRPPRC	9	10	90.00
DHX9	6	6	100.00
SMC2	8	8	100.00

IARS	7	7	100.00
CAND1	5	5	100.00

Mass spectrum analysis of the lower band.

Protein	Unique peptide	Total peptides	% peptide ratio
ACTB	11	20	55.00
**TFAP2A**	**8**	**9**	**88.89**
GOT2	6	6	100.00
LRPAP1	2	2	100.00

table S2. Primers for real-time reverse transcription PCR analysis.

Gene	Forward	Reverse
Gapdh	CCTCCTGCAGACAGACGTAA	AGCATCGACCAGTGCTACAG
Ciita	TGGGATCTTCCAGCGGAAGC	ACAACAGGGCTGTGACTATAGC
Alp	CCAACTCTTTTGTGCCAGAGA	GGCTACATTGGTGTTGAGCTTTT
Col1a1	GCTCCTCTTAGGGGCCACT	CCACGTCTCACCATTGGGG
Bglap	CTCTGACCTCACAGATGCCA	TTATTGCCCTCCTGCTTGGA
Trap	CACTCCCACCCTGAGATTTGT	CATCGTCTGCACGGTTCTG
Calcr	GAGGTTCCTTCTCGTGAACAG	AGTCAGTGAGATTGGTAGGAGC
Ctsk	GAAGAAGACTCACCAGAAGCAG	TCCAGGTTATGGGCAGAGATT
Il6	TAGTCCTTCCTACCCCAATTTCC	TTGGTCCTTAGCCACTCCTTC
Il8	CAAGGCTGGTCCATGCTCC	TGCTATCACTTCCTTTCTGTTGC
Il17	TTTAACTCCCTTGGCGCAAAA	CTTTCCCTCCGCATTGACAC
Il1β	GCAACTGTTCCTGAACTCAACT	ATCTTTTGGGGTCCGTCAACT
Tnfα	CCCTCACACTCAGATCATCTTCT	GCTACGACGTGGGCTACAG
Tgfβ	CTCCCGTGGCTTCTAGTGC	GCCTTAGTTTGGACAGGATCTG
Igf1	CTGGACCAGAGACCCTTTGC	GGACGGGGACTTCTGAGTCTT
Tnfsf11	CAGCATCGCTCTGTTCCTGTA	CTGCGTTTTCATGGAGTCTCA
Sost	AGCCTTCAGGAATGATGCCAC	CTTTGGCGTCATAGGGATGGT
Dmp1	CATTCTCCTTGTGTTCCTTTGGG	TGTGGTCACTATTTGCCTGTG
Fgf23	ATGCTAGGGACCTGCCTTAGA	AGCCAAGCAATGGGGAAGTG
Dkk1	CTCATCAATTCCAACGCGATCA	GCCCTCATAGAGAACTCCCG
Opg	ACCCAGAAACTGGTCATCAGC	CTGCAATACACACACTCATCACT
Mepe	GTCTGTTGGACTGCTCCTCTT	CACCGTGGGATCAGGATACA

M-csf	ATGAGCAGGAGTATTGCCAAGG	TCCATTCCCAATCATGTGGCTA
Sfrp1	TCTAAGCCCCAAGGTACAACC	GCTTGCACAGAGATGTTCAATG
Clec9a	GAAGTGCCAATCCCCTAGCAA	CAGTCACTACCTGAATGGAGAGA
Riken	CTCCAGTTGCTGTGGGAATG	CTGAATAGCCAGCCAAGCAC
Adgrg5	GATTCCACCACACGTATCCAC	CAGTGCGTAGTGTAGGACAGC
Ciita Type I	CAGGGACCATGGAGACCATAGT	CAGGTAGCTGCCCTCTGGAG
Ciita Type III	GGTTCCTGGCCCTTCTGG	ATCCATGGTGGCACACAGACT
Ciita Type IV	CAGCACTCAGAAGCACGGG	ATCATGGTGGCACACAGACT
TP	ACAGGAGGCACCTTGGATAA	GCTCACTCTGACCCACGATA
RANKL	ATCGTTGGATCACAGCACAT	TTATGGGAACCAGATGGGAT
SOST	ACACAGCCTTCCGTGTAGTG	GGTTCATGGTCTTGTTGTTCTCC
GAPDH	CTGGGCTACACTGAGCACC	AAGTGGTCGTTGAGGGCAATG

table S3. Primers for constructing vector constructs in luciferase assay.

Name	Forward	Reverse
Luc-Tnfsf11	CATCTCGAG GGAGGCAGAGAGGCAGAGACA	CAGAAGCTT CTCCCTTCCTCGGCCACCCA
Luc-Tnfsf11-Δ1	CATCTCGAG GCCTTCACCAGCTGAGCCGTC	CAGAAGCTT CTCCCTTCCTCGGCCACCCA
*Luc-Tnfsf11-*mut	CAGAAGAATCCTCATCCTTT TTATCCATCATGTGCGCAAG CACGCACAC	GTGTGCGTGCTTGCGCACAT GATGGATAAAAAGGATGAGG ATTCTTCTG
Luc-Sost	CATCTCGAG AGGCGGACCACAGCCTCCTT	CAGAAGCTT CCCTCTGCCTCATGCCAGCC
Luc-Sost-Δ1	CATCTCGAG GCCTGGTGGTTGCCTCTCCC	CAGAAGCTT CCCTCTGCCTCATGCCAGCC
Luc-Sost-Δ2	CATCTCGAG TGCCCGTGGGAGGTACTGGG	CAGAAGCTT CCCTCTGCCTCATGCCAGCC
*Luc-Sost-*mut1	GGCCCACAGTTTGTTTTGAG AATGATTGATAGGCCCAGGG AGCCAG	CTGGCTCCCTGGGCCTATCAA TCATTCTCAAAACAAACTGTG GGCC
Luc-*Sost-*mut2	CTTGGCTTTTAATTGTCTGG CTTACTGATCCCCTCAGGCA TTCTCAAAACA	TGTTTTGAGAATGCCTGAGG GGATCAGTAAGCCAGACAAT TAAAAGCCAAG

table S4. Primers for ChIP-PCR.

Genes	Forward	Reverse
Irf1	TGCAGAAAGAGGGGGACGGT	GCGGCGAAGGGGAAGTACAA
Ciita	GCTGGAGCTCACCATGTCCC	TGGGCCAAATTGGGTGACCA
Tnfsf11	CCAACCACTGGACCCAACCC	AGATGGCAGGGTACCCCAGG
Sost	AAACCACAGCCTGACTGCCC	GTGTGCTTAGCGTCAGCCCT
