# Supplementary material for: Osteocyte CIITA aggravates osteolytic bone lesions in myeloma
Source: Nat Commun. 2022 Jun 27;13:3684. doi: 10.1038/s41467-022-31356-7 (PMC9237076; doi:10.1038/s41467-022-31356-7)
Supplement: Supplementary file 2 — Reporting Summary [file 41467_2022_31356_MOESM2_ESM.pdf]

nature research

Corresponding author(s): Jing Yang

Last updated by author(s): 06/06/2022

Reporting Summary

Nature Research wishes to improve the reproducibility of the work that we publish. This form provides structure for consistency and transparency in reporting. For further information on Nature Research policies, see our Editorial Policies and the Editorial Policy Checklist.

Statistics

For all statistical analyses, confirm that the following items are present in the figure legend, table legend, main text, or Methods section.

n/a	Confirmed	
☐	☒	The exact sample size (n) for each experimental group/condition, given as a discrete number and unit of measurement
☐	☒	A statement on whether measurements were taken from distinct samples or whether the same sample was measured repeatedly
☐	☒	The statistical test(s) used AND whether they are one- or two-sided<br>*Only common tests should be described solely by name; describe more complex techniques in the Methods section.*
☒	☐	A description of all covariates tested
☒	☐	A description of any assumptions or corrections, such as tests of normality and adjustment for multiple comparisons
☐	☒	A full description of the statistical parameters including central tendency (e.g. means) or other basic estimates (e.g. regression coefficient) AND variation (e.g. standard deviation) or associated estimates of uncertainty (e.g. confidence intervals)
☐	☒	For null hypothesis testing, the test statistic (e.g. F, t, r) with confidence intervals, effect sizes, degrees of freedom and P value noted<br>*Give P values as exact values whenever suitable.*
☒	☐	For Bayesian analysis, information on the choice of priors and Markov chain Monte Carlo settings
☒	☐	For hierarchical and complex designs, identification of the appropriate level for tests and full reporting of outcomes
☒	☐	Estimates of effect sizes (e.g. Cohen's d, Pearson's r), indicating how they were calculated

Our web collection on statistics for biologists contains articles on many of the points above.

Software and code

Policy information about availability of computer code

Data collection	Flow cytometry:<br>BD LSRFortessa flow cytometer (BD Biosciences)was used to collect flow cytometry data.<br>Mass spectrometry:<br>The tryptic peptides were then analyzed using a nano-LC/MS/MS (Thermo Fisher Scientific) coupled with an 1100 HPLC (Agilent Technologies). The MS/MS spectra were searched using the SEQUEST software program with the BioWorks Browser (version 3.3.1; Thermo Fisher Scientific) against the NCBI database.<br>RNA seq analysis:<br>RNA-seq libraries were synthesized according to the manufacturer's instructions (Illumina). Pooled libraries were quantified using the KAPA Library Quantification Kit (Kapa Biosystems), examined for size distribution using the Fragment Analyzer (Advanced Analytical), and, using the 76-bp paired-end format, sequenced in four lanes of the Illumina HiSeq 4000 Sequencer.
Data analysis	GraphPad Prism (version 9) or SPSS software program (version 10.0; IBM Corporation) were used for bar graphs output and statistic analysis. FlowJo (version 10) was used for flow cytometry data analysis. TRAP and toluidine blue staining were analyzed using the BIOQUANT OSTEO (version 18.2.6) software program (BIOQUANT Image Analysis Corporation). Mouse femurs were scanned with Scanco micro–computed tomography (µCT)-40 system (Scanco Medical) and analyzed with Scanco (version 6.1) or Microview (version 2.5, Parallax Innovations) software.

For manuscripts utilizing custom algorithms or software that are central to the research but not yet described in published literature, software must be made available to editors and reviewers. We strongly encourage code deposition in a community repository (e.g. GitHub). See the Nature Research guidelines for submitting code & software for further information.

Data

Policy information about availability of data

All manuscripts must include a data availability statement. This statement should provide the following information, where applicable:

- Accession codes, unique identifiers, or web links for publicly available datasets
- A list of figures that have associated raw data
- A description of any restrictions on data availability

All data associated with this study can be found in the paper or the supporting documents. Uncropped scans of immunoblots and gels are provided in the Supplementary Information. Source data are provided with this paper. The RNA-seq data generated in this study is available at GEO database (GSE200987).

Field-specific reporting

Please select the one below that is the best fit for your research. If you are not sure, read the appropriate sections before making your selection.

☒ Life sciences ☐ Behavioural & social sciences ☐ Ecological, evolutionary & environmental sciences

For a reference copy of the document with all sections, see nature.com/documents/nr-reporting-summary-flat.pdf

Life sciences study design

All studies must disclose on these points even when the disclosure is negative.

Sample size	Sample sizes were determined and based on previous publications of similar studies and previous experiences.
Data exclusions	No data were excluded from the analysis.
Replication	Experimental findings were reliably reproduced in multiple mice or cell lines in each study as replicates.
Randomization	All the samples used in the study were randomly allocated.
Blinding	For cell-based experiments, investigators were not blinded during data collection or analysis. However, controls and samples were analyzed in the same manner. Animal experiments and mass spectrometry analysis were performed in a blind manner to group allocation during data collection.

Reporting for specific materials, systems and methods

We require information from authors about some types of materials, experimental systems and methods used in many studies. Here, indicate whether each material, system or method listed is relevant to your study. If you are not sure if a list item applies to your research, read the appropriate section before selecting a response.

Materials & experimental systems

n/a	Involved in the study	
☐	☒	Antibodies
☐	☒	Eukaryotic cell lines
☒	☐	Palaeontology and archaeology
☐	☒	Animals and other organisms
☐	☒	Human research participants
☒	☐	Clinical data
☒	☐	Dual use research of concern

Methods

n/a	Involved in the study	
☒	☐	ChIP-seq
☐	☒	Flow cytometry
☒	☐	MRI-based neuroimaging

Antibodies

Antibodies used

The following antibodies were used:
WESTERN BLOT:
CIITA antibody (3793, Cell Signaling Technology, 1:1000),
GAPDH antibody (5174, Cell Signaling Technology, 1:5000),
Phospho-p44/42 MAPK antibody (Erk1/2) (Thr202/Tyr204) (4370, Cell Signaling Technology, 1:1000),
p44/42 MAPK antibody (Erk1/2) (4695, Cell Signaling Technology, 1:2000),
Phospho-Akt antibody (Ser473) (4058, Cell Signaling Technology, 1:1000)
Akt Antibody (9272, Cell Signaling Technology, 1:2000),
Phospho-Syk (Tyr525/526) antibody (2710, Cell Signaling Technology, 1:1000),
Syk Antibody (2712, Cell Signaling Technology, 1:1000),

Phospho-STAT1 antibody (Tyr701) (9167, Cell Signaling Technology, 1:1000),
STAT1 Antibody (14994, Cell Signaling Technology, 1:1000),
Phospho-JAK1 antibody (Tyr525/526) (74129, Cell Signaling Technology, 1:1000),
JAK1 Antibody (3332, Cell Signaling Technology, 1:1000),
Histone H3 antibody (4499, Cell Signaling Technology, 1:5000),
IRF1 antibody (8478, Cell Signaling Technology, 1:1000),
Histone H3 (acetyl K9 + K14 + K18 + K23 + K27) antibody (ab47915, abcam, 1:1000),
Histone H4 (acetyl K5 + K8 + K12 + K16) antibody [EPR16606] (ab177790, abcam, 1:1000),
Acetyl-Histone H3 (Lys14) (D4B9) antibody (7627, Cell Signaling Technology, 1:1000),
Acetyl-Histone H3 (Lys27) (D5E4) antibody (8173, Cell Signaling Technology, 1:1000),
AP-2α (C83E10) antibody (3215, Cell Signaling Technology, 1:1000),
Rabbit (DA1E) mAb IgG Isotype Control (3900, Cell Signaling Technology, 1:100).

FLOW CYTOMETRY:
APC anti-mouse/human CD44 Antibody (clone:IM7, BioLegend, 103011, 1:200),
APC anti-mouse CD105 Antibody (clone: MJ7/18, BioLegend, 120413, 1:200),
Mouse DMP-1 antibody (R&D Systems, AF4386, 1:200)
Mouse sclerostin antibody (Clone: 248121, R&D Systems, MAB1589, 1:200)
Mouse osteocalcin (E6) antibody (Santa Cruz, sc376835, 1:100)
Mouse COL1A1 antibody (clone: EPR24331-53, abcam, ab270993, 1:300)
Goat anti-Mouse IgG (H+L) Cross-Adsorbed Secondary Antibody, APC (Thermo Fisher scientific, A-865, 1:300),
APC-H7, Mouse IgG1, Isotype Control (BD Biosciences, 560167, 1:200)

IMMUNOFLUORESCENT STAINING:
Anti-podoplanin Antibody (8.1.1) (Santa Cruz, sc-53533, 1:50)
Alexa Fluor 488-labeled goat anti-hamster IgG (H+L) Cross-Adsorbed Secondary Antibody (Thermo Fisher, A-21110, 10 µg/mL)

IP
Pierce™ Anti-c-Myc Agarose (Thermo Fisher Scientific, 20169)

Validation

All antibodies were used in accordance to the manufacturer guidelines and confirmed by the manufacturers and/or to have previously been documented in the literature through CiteAb (www.citeab.com).

WESTERN BLOT:
CIITA (Cell Signaling Technology) antibody was validated by western blot analysis of extracts from COS cells, mock transfected or transfected with human CIITA.
GAPDH (Cell Signaling Technology) antibody was validated by western blot analysis of extracts from various cell lines (HeLa, NIH/3T3, C6, COS-7).
Phospho-p44/42 MAPK (Erk1/2) (Thr202/Tyr204) (Cell Signaling Technology) antibody was validated by western blot analysis of extracts from COS cells, untreated or treated with either U0126 (10 µM for 1 hour) or TPA (200 nM for 10 minutes).
p44/42 MAPK (Erk1/2) (Cell Signaling Technology) antibody was validated by western blot analysis of extracts from HeLa, NIH/3T3 and C6 cells.
Phospho-Akt (Ser473) (Cell Signaling Technology) antibody was validated by western blot analysis of extracts from untreated or PDGF-treated NIH/3T3 cells, pretreated with wortmannin and/or rapamycin.
Akt Antibody (Cell Signaling Technology) was validated by western blot analysis of extracts from CHO cells, transfected with non-targeted control or Akt siRNA.
Phospho-Syk (Tyr525/526) (Cell Signaling Technology) antibody was validated by western blot analysis of extracts from Ramos cells, untreated or treated with anti-IgM.
Syk Antibody (Cell Signaling Technology) antibody was validated by western blot analysis of extracts from Jurkat and THP1 cells, and un-transfected or Syk cDNA-transfected DT40 cells.
Phospho-STAT1 (Tyr701) (Cell Signaling Technology) antibody was validated by western blot analysis of extracts from HeLa cells untreated or treated with interferon-α (IFN-α).
STAT1 (Cell Signaling Technology) antibody was validated by western blot analysis of extracts from A549 cells or STAT1 knock-out cells.
Phospho-JAK1 (Tyr525/526) (Cell Signaling Technology) antibody was validated by western blot analysis of extracts from U266 or Jurkat cells, serum-starved overnight followed by treatment with Human Interferon-α1 (hIFN-α1, 10 ng/ml, 15 min) or Human Interleukin-4 (hIL-4, 10 ng/ml, 10 min).
JAK1 (Tyr525/526) (Cell Signaling Technology) antibody was validated by western blot analysis of extracts from CTLL-2 and BaF3 cells.
Histone H3 (Cell Signaling Technology) antibody was validated by western blot analysis of extracts from
various cell lines (HeLa, NIH/3T3, C6, COS-7).
IRF1 (8478, Cell Signaling Technology, 1:1000) antibody was validated by western blot analysis of extracts from HeLa cells, untreated or treated with Human Interferon-γ (hIFN-γ) (100 ng/ml, 4 hours).
Histone H3 (acetyl K9 + K14 + K18 + K23 + K27) antibody (abcam) recognizes histone H3 acetylated at lysines 9, 14, 18, 23 or 27 as confirmed by dot blot with non-modified histone H3 peptide or peptides. No reaction with non-modified histone H3 peptide as tested by dot blot.
Histone H4 (acetyl K5 + K8 + K12 + K16) antibody [EPR16606] (abcam) was validated by western blot analysis of extracts from HeLa cells treated with Sodium butyrate extract lysates; NIH/3T3 cells treated with Trichostatin A.
Acetyl-Histone H3 (Lys14) (D4B9) antibody (Cell Signaling Technology) was validated by western blot analysis of extracts from HeLa, C2C12, and COS-7 cells, untreated or treated with Trichostatin A (1 µM, 18 hours),

Acetyl-Histone H3 (Lys27) (D5E4) antibody (Cell Signaling Technology) was validated by western blot analysis of extracts from HeLa and C2C12 cells, untreated or treated with Trichostatin A (1 µM, 18 hours),
AP-2α (C83E10) antibody (3215, Cell Signaling Technology, 1:1000) was validated by western blot analysis of extracts from various cell lines (MCF7, HeLa, A204, KNRK, COS-7).

FLOW CYTOMETRY:
FITC Mouse Anti-Human CD44 (BD Biosciences) was validated by flow cytometric analysis of CD44 expression on human peripheral blood lymphocytes.
APC Mouse Anti-Human CD105 (BD Biosciences) was validated by flow cytometric analysis of CD44 expression on U937 cells.
Mouse DMP-1 Antibody (R&D) was validated by western blot analysis of extracts from mouse myeloma cell line NS0-derived recombinant mouse DMP-1.
Mouse sclerostin antibody (R&D) was validated by western blot analysis of extracts from mouse myeloma cell line NS0-derived recombinant mouse SOST.
Mouse osteocalcin antibody (Santa Cruz) was validated by western blot analysis of mouse recombinant osteocalcin fusion protein.
Mouse COL1A1 antibody (abcam) was validated by western blot analysis of extracts from NIH/3T3 cells.

IMMUNOFLUORESCENT STAINING:
Anti-podoplanin Antibody (8.1.1) (Santa Cruz) was validated by western blot analysis of tissue extracts from mouse lymph, kidney and thymus as well as extracts from B4 and C3H cells.

Eukaryotic cell lines

Policy information about cell lines

Cell line source(s)	The myeloma cell line ARP-1 was established and provided by the University of Arkansas for Medical Sciences. Murine myeloma Vk*MYC cell line (Vk12598) was established and provided by the Mayo Clinic. HEK293T, Raw264.7, and MC3T3-E1 cells were purchased from the American Type Culture Collection. MLO-Y4 and MLO-A5 cells were purchased from Kerafast, Inc.
Authentication	Authentication of human cell line identity was confirmed using short tandem repeat (STR) fingerprinting. Murine myeloma cell lines will be validated by examination the pattern of M-spike using serum protein electrophoresis as published by the provider.
Mycoplasma contamination	All cell lines tested negative for mycoplasma contamination.
Commonly misidentified lines (See ICLAC register)	No commonly misidentified lines were used in this study.

Animals and other organisms

Policy information about studies involving animals; ARRIVE guidelines recommended for reporting animal research

Laboratory animals	Male or female aged 6-8 weeks of CB.17 SCID or C57BL/6 mice purchased from Harlan Laboratories, Dmp-1-cre/ERT2 mice purchased from The Jackson Laboratory, and homozygous Ciita2flox/flox mice purchased from Charles River Laboratories, were maintained in American Association for Laboratory Animal Science–accredited facilities. All mice were maintained on a standard light and dark cycle with food and water. The animal room had a controlled temperature (23-24 °C), humidity (60.5%). Cage are cleaned every 3-4 days and supplies of water and food are checked daily.
Wild animals	This study did not involve wild animals.
Field-collected samples	No field-collected samples were used in the study.
Ethics oversight	All in vivo mouse studies were approved by the UT MD Anderson Institutional Animal Care and Use Committee.

Note that full information on the approval of the study protocol must also be provided in the manuscript.

Human research participants

Policy information about studies involving human research participants

Population characteristics	Bone marrow biopsies from myeloma patients (n=15) were used;<br>Bone marrow serum of myeloma patients (n=15) were used;<br>MSCs were obtained from the bone marrow of healthy donors (n=5);
Recruitment	This study didn't recruit any human subjects. All patient samples were retrospectively obtained through the Myeloma Tissue Bank at The University of Texas MD Anderson Cancer Center or Xiamen University.
Ethics oversight	This study was approved by the MD Anderson Institutional Review Board (PA12-0034) and the Ethics Committee of Xiamen University. Informed consent was obtained from study participant and collection of patient samples was conducted by both institutions in accordance with the criteria set by the Declaration of Helsinki.

Note that full information on the approval of the study protocol must also be provided in the manuscript.

Flow Cytometry

Plots

Confirm that:

- [x] The axis labels state the marker and fluorochrome used (e.g. CD4-FITC).
- [x] The axis scales are clearly visible. Include numbers along axes only for bottom left plot of group (a 'group' is an analysis of identical markers).
- [x] All plots are contour plots with outliers or pseudocolor plots.
- [x] A numerical value for number of cells or percentage (with statistics) is provided.

Methodology

Sample preparation	For CD44 and CD105 analysis, cells were fixed and stained with antibodies measured by a BD LSRFortessa flow cytometer (BD Biosciences). For DMP-1, sclerostin, osteocalcin and col1a1 analysis, fixed osteocytes were permeabilized and blocked in 1% BSA in 1 x PBST for 30 min at 37°C. Then the cells were incubated with DMP-1, sclerostin, osteocalcin or col1a1 antibody (R&D) and subsequent secondary antibodies conjugated with Alexa Fluor 488 (Invitrogen) or goat IgG isotype control (Novus Biologicals), and measured by a BD LSRFortessa flow cytometer (BD Biosciences).
Instrument	BD LSRFortessa flow cytometer (BD Biosciences).
Software	FlowJo (version 10) was used for flow cytometric data analysis.
Cell population abundance	Not applicable (all cells used).
Gating strategy	Signal cells were gated according to forward-scatter and side-scatter profiles.

- [ ] Tick this box to confirm that a figure exemplifying the gating strategy is provided in the Supplementary Information.
